# Hepatoprotective potential of a novel quinazoline derivative in thioacetamide-induced liver toxicity

**DOI:** 10.3389/fphar.2022.943340

**Published:** 2022-09-20

**Authors:** Suzy Salama, Chin Siang Kue, Haryanti Mohamad, Fatima Omer, Mohamed Yousif Ibrahim, Mahmood Abdulla, Hapipah Ali, Abdalbasit Mariod, Soher Nagi Jayash

**Affiliations:** ^1^ Indigenous Knowledge and Heritage Center, Ghibaish College of Science and Technology, Ghibaish, Sudan; ^2^ Faculty of Health and Life Sciences, Management and Science University, Shah Alam, Selangor, Malaysia; ^3^ Animal Experimental Unit, Faculty of Medicine, University of Malaya, Kuala Lumpur, Malaysia; ^4^ Department of Chemistry and Biology, Faculty of Education-Hantoub, University of Gezira, Gezira, Sudan; ^5^ Faculty of Pharmacy, Elrazi University, Khartoum, Sudan; ^6^ Faculty of Pharmacy, University of Sinnar, Sinja, Sudan; ^7^ Department of General Biology, College of Science, Cihan University-Erbil, Erbil, Kurdistan, Iraq; ^8^ Department of Chemistry, Faculty of Science, University of Malaya, Kuala Lumpur, Malaysia; ^9^ Faculty of Science & Arts, University of Jeddah, Alkamil, Kingdom of Saudi Arabia; ^10^ School of Dentistry, University of Birmingham, Birmingham, United Kingdom

**Keywords:** quinazoline, indocyanine green-imaging, oxidative stress, liver fibrosis, liver toxicity

## Abstract

**Purpose:** The compound quinazoline Q-Br, 3-(5-bromo-2-hydroxybenzylideneamino)-2-(5-bromo-2 hydroxyphenyl) 2,3-dihydroquinazoline-4(1H)-one (Q-Br) was evaluated for its antioxidant capacity and potential hepatoprotectivity against sub-chronic liver toxicity induced by thioacetamide in rats.

**Materials and Methods:** Rats were assigned into five groups; healthy (normal) and cirrhosis control groups were given 5% Tween 20 orally, the reference control group was given a Silymarin dose of 50 mg/kg, and low-dose Q-Br and high-dose Q-Br groups were given a daily dose of 25 mg/kg and 50 mg/g Q-Br, respectively. Liver status was detected via fluorescence imaging with intravenous injection of indocyanine green (ICG) and a plasma ICG clearance test. Liver malondialdehyde (MDA), catalase (CAT), superoxide dismutase (SOD), and glutathione peroxidase (GPx) were also tested. The degree of fibrosis was determined histologically by hematoxylin and eosin and Masson’s Trichrome staining. The immunohistochemistry of liver tissue inhibitor of metalloproteinase (TIMP-1), matrix metalloproteinase (MMP-2), and alpha-smooth muscle actin (α-SMA) was performed.

**Results:** Q-Br recorded mild antioxidant capacity, dose-dependent improvement in the liver status, and inhibition of oxidative stress compared to cirrhosis control. Histopathology notified a remarkable reduction in the degree of fibrosis. Immunohistochemistry revealed an obvious low expression of MMP-2 and α-SMA along with a higher expression of TIMP-1 in Q-Br- and Silymarin-treated livers.

**Conclusion:** Q-Br treatment altered the course of toxicity induced by thioacetamide suggesting significant hepatoprotective potential of Q-Br treatment.

## 1 Introduction

Liver fibrosis denotes a pathologically abnormal state of hepatocytes, which may originate from genetic, chemical, or environmental factors (Delire et al., 2015). Regardless of its origin, the condition is accompanied by an inflammatory response with abundant deposition of the extracellular matrix (ECM) including fibrillar collagen (O'Connell and Rushworth, 2008; Salama et al., 2013). During fibrosis, the balance between degradation and regeneration of the connective tissue components is disturbed (Czechowska et al., 2015). Liver injury starts as inflammation and develops as an abnormal wound healing process leading to cirrhosis and ends with hepatocellular carcinoma in the majority of liver diseases (Van der Meer et al., 2015). As estimated by some studies, the cases of cirrhosis-mediated hepatocellular carcinoma are 80% (Llovet and Bruix, 2003). Additionally, compensated cirrhotic livers recorded high mortality in patients with hepatocellular carcinoma (Fattovich et al., 2004). With the hope of anti-fibrotic therapy to revive the normal liver architecture and function, liver fibrosis can be considered a bidirectional process (Friedman, 2010). Thioacetamide (TAA) is a carcinogenic chemical that is used by researchers to induce liver damage in chronic and acute experiments. Its mechanism of damage depends mainly on oxidative stress (Staňková et al., 2010).

Peroxidation of the hepatocellular lipid layer and disturbance in liver cell functions were considered the main features of oxidative stress in liver injury (Mostafa et al., 2021). The mechanism of deterioration in the biochemical reactions inside liver cells damaged by thioacetamide and its oxidative stress leads to massive inflammatory responses and necrosis of hepatocytes (Chang et al., 2021).

Recently, many studies have been conducted on synthetic compounds in trying to find convenient therapies to stop or at least inhibit the progress of liver fibrosis such as barbituric acid derivatives (Wang et al., 2020) and glycerrhetinic acid derivatives (Zhang et al., 2020). To our knowledge, quinazoline and its derivatives exhibited pharmaceutical efficacies as a chemotherapeutic agent in cancer (Bansal and Malhotra, 2020) and promising therapy against coronavirus (COVID-19). Borik and Hussein (2022a) have recently published that quinazoline derivatives showed very good antioxidant and anti-inflammatory activities via their molecular docking study and their study of the relationship between structure and activity of quinazoline compound. Furthermore, Lv et al. (2020) reported that synthetic phenyl chloromethine-quniazoline derivative has proved induction of apoptosis in *in vivo* and *in vitro* hepatic cancer cells. In this context, this research was conducted to estimate the hepatoprotective activity of a quinazoline Schiff base compound (Q-Br) against sub-chronic thioacetamide toxicity in rats. To our knowledge, no experimental study was conducted on the novel quinazoline derivative Q-Br. Therefore, the present study was designed to evaluate the anti-oxidative and antifibrotic mechanism of Q-Br in the liver tissues of rats through the histological study of the animals’ liver tissues treated with low- and high-doses of Q-Br compound using Silymarin as a standard drug.

## 2 Materials and Methods

### 2.1 Synthesis and compound preparation

**FIGURE 1:**
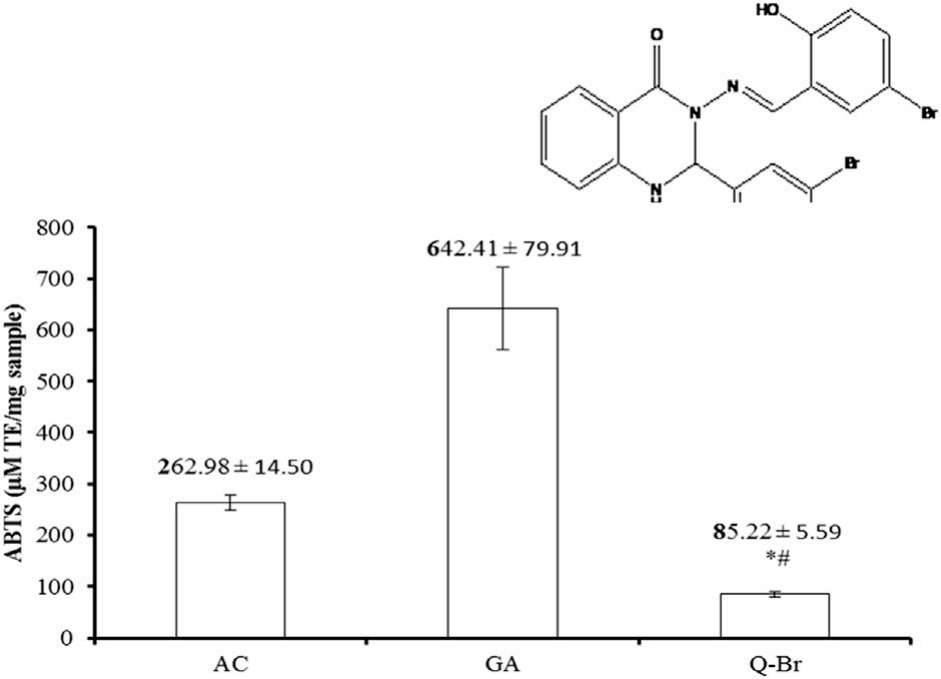
ABTS scavenging capacity of Q-Br compared to the standard ascorbic acid (AC) and gallic acid (GA). The data are displayed as mean ± SD (*n* = 3) and *p* ≤ 0.05 was considered significant. *p < 0.01 vs. AC and ^#^ *p* < 0.001 vs. GA. The structure of the synthetic quinazoline Schiff base compound, 3-(5-Bromo-2-hydroxybenzylideneamino)-2-(5-bromo-2 hydroxyphenyl)-2,3-dihydroquinazoline-4(1H)-one (Q-Br) is indicated above Q-Br bar.

The compound *3-(5-Bromo-2-hydroxybenzylideneamino)-2-(5-bromo-2 hydroxyphenyl)-2,3-dihydroquinazoline-4(1H)-one* which in short named as Q-Br (Figure 1) is quinazoline Schiff base compound and is prepared as previously mentioned (Faraj et al., 2014) in the Department of Chemistry, Faculty of Science, University of Malaya. The compound’s purity was confirmed using thin layer chromatography (TLC), Carbon, Hydrogen, and Nitrogen (CHN) analysis, and 1H and 13C-Nuclear Magnetic Resonance (NMR) spectroscopy. Based on the acute toxicity test conducted previously on the said compound (Faraj et al., 2014), the doses 25 mg/kg and 50 mg/kg were chosen as 1/10 of the safe fixed dose tested (250 mg/kg) and double the one 10th dose. Before use, both doses of Q-Br were prepared by dissolving in 5% Tween 20.

### 2.2 Assessment of the azino-bis 3-ethylbenzthiazoline-6-sulfonic scavenging activity of Q-Br

The antioxidant capacity of Q-Br was measured by its scavenging power to the cation radical 2,2′-azino-bis 3-ethylbenzthiazoline-6-sulfonic (ABTS^+^) following the protocol of the Zen-Bio kits’ manuals (Cat # ZBM0034.0, China). Briefly, ABTS^+^ radical was produced by the oxidation of ferryl myoglobin radical to ABTS compound and the record of the spectrophotometer was read at 405 nm. The antioxidant power of Q-Br could be detected by its inhibition of the green-colored ABTS^+^ radical formed and compared to similar concentrations of ascorbic acid (AC) and gallic acid (GA).

### 2.3 *In vivo* hepatoprotective evaluation of Q-Br

#### 2.3.1 Animals

Thirty male Sprague Dawley rat species having an age of 6–8 weeks were used in the present experiment. The animals were maintained following the regulations of the “Guide for the Care and Use of Laboratory Animals Eighth Edition” by the National Research Council published by National Academies Press. The animal use protocol was approved by the Faculty of Medicine Institutional Animal Care and Use Committee (FOM IACUC) under Ethic Reference no: 2014–10-14/CHEMS/R/SMSF. All rats were kept at 56–70% humidity and 19–21°C, given reverse osmosis and autoclavable maintenance diet, and exposed to 12 h of light/dark cycles.

### 2.4 Design of the experiment

Following acute administration of thioacetamide (TAA), centrilobular necrosis is induced, while long-term administration of TAA toxicity can produce proliferation in the bile duct and initiate liver cirrhosis that is identical to the liver histology provoked by viral hepatitis infection (Constantinou et al., 2007). Silymarin is a complex prepared purely from *Silybum marianum* plant seeds and generally used as a helpful treatment for liver diseases such as fibrosis, hepatic diseases, and infiltration of fatty acids due to toxins and alcohol (Schrieber et al., 2008). Therefore, the positive results of Q-Br therapy in liver damage induced by the insult TAA were compared with the protection provided by the reference drug, Silymarin in this research. Rats were separated into five groups; the normal control group (5% Tween 20, 5 ml/kg), the cirrhosis control group (5% Tween 20.5 ml/kg), the reference control group (Silymarin 50 mg/kg)with, LD Q-Br (25 mg/kg) and HD Q-Br (50 mg/kg). The control groups were gavaged with 5% Tween 20 at a dose of 5 ml/kg because Silymarin and Q-Br doses were prepared by dissolving them in 5% Tween 20 as previously performed in our experiment.

The rats were all daily orally gavaged with the doses mentioned. With the exception of normal control rats, all the animals were given an intra-peritoneal injection dose of TAA 200 mg/kg three times weekly for 15 weeks. The body weight of all the animals was recorded on the first day of the experiment and monitored weekly throughout the whole study.

At the end of the experiment and 24 h after the last treatment with TAA, the animals fasted overnight. The rats were anesthetized using an intramuscular dose of ketamine and xylazine cocktail (xylazine 3 mg/kg and ketamine 30 mg/kg) followed by weighing and exposing their tails to infrared light for 10 min to dilate the tail vein. A bolus of indocyanine green (ICG) at a dose of 0.5 mg/kg was then administered via intravenous injection into the tail vein of each rat (3 rats/group) (Gupta et al., 2012). A volume of 0.3–0.5 ml of blood was taken from each rat’s vein at 5, 10, 15, and 20 min post-ICG injection into EDTA blood collecting tubes for performing the plasma indocyanine green clearance assay. Another amount of blood was collected from each rat (6 rats/group) by the cardiac puncture method in properly labeled gel collecting blood tubes. Thereafter, all the rats were euthanized using ketamine and xylazine overdose by mixing xylazine (10 mg/kg) and ketamine (80 mg/kg) for postmortem study.

### 2.5 Biochemistry analysis

The potential effect of the Q-Br treatment on TAA-toxicity was estimated through the liver and renal function tests, the blood of each animal was collected in properly labeled gel collecting blood tubes and centrifuged after adjusting the machine at 3,500 rpm for 10 min at 4°C. The serum collected was sent to the Central Diagnostic Laboratory of the Medical Center of University Malaya for evaluation of serum level of alkaline phosphatase (AP), alanine aminotransferase (ALT), aspartate aminotransferase (AST), Lactate dehydrogenase (LDH), gamma glutamyl transaminase (GGT), and bilirubin.

### 2.6 Liver bioimaging

Following euthanization, the livers of animals were excised carefully and weighed. The images of livers which were collected from three rats/group were captured using *in vivo* Ms Fx Pro (Carestream Molecular Imaging, Woodbridge, CT). The excitation filter was adjusted at 510 nm, while the emission filter was set at 550 nm with an exposure time of 5 s. The intensity of fluorescence was quantitatively measured using imaging program 5.0 (Carestream Molecular Imaging, Woodbridge, CT) (Kue et al., 2015).

### 2.7 Plasma indocyanine green clearance assay

As per previous studies, researchers used the disappearance rate of plasma test of ICG in detecting liver function because the ICG assay is a sensitive test in detecting the prognosis of the cirrhotic liver (Faybik and Hetz, 2006; Quintero et al., 2014). The blood collected for this purpose was centrifuged after adjusting the centrifuge speed at 3,000 rpm for 10 min at 4°C and the plasma was collected in precisely labeled tubes. The obtained plasma samples were assayed immediately using an indocyanine green clearance assay following the procedure of Jaensch *et al.* (Jaensch et al., 2000) with minor modification. In brief, the blood collected from animals was 0.3–0.5 ml instead of 0.3–0.5 ml collected in Jaensch’s study, blood was collected 3, 6, and 9 min after ICG injection instead of 5, 10, 15, and 20 min after ICG injection used in Jaensch’s study and centrifugation was performed at 3,000 rpm for 5 min instead of 10 min in Jaensch’s study. ICG (Sigma-Aldrich, United States) was reconstituted in sterile normal saline (0.5 mg/ml) and then serially diluted (5, 2.5, 1.25, and 0.625 μg/ml) from the stock solution using normal rat plasma. A volume of 100 µL of the standard/plasma was collected from each rat at 5, 10, 15, and 20 min and was sampled into a 96-well plate in triplicate and the record of the spectrophotometer was read at 805 nm as followed by protocol. The concentration of ICG in the plasma of each rat was calculated from the standard curve produced at 805 nm.

### 2.8 Assessment of liver malondialdehyde level and antioxidant enzymes

For studying the biochemistry of the liver tissue homogenate, a piece of 500 mg from each rat’s liver was cut and homogenized in prepared phosphate-buffered saline (PBS, 5 ml, pH 7.2) at 10,000 ₓg for 15 min at 4°C. The collected supernatant from each animal was aliquoted and kept in labeled vials at −80°C till further assessment. Hepatic protein level was measured using the Bradford method (Bradford, 1976). Lipid peroxidation grade imposed by TAA intoxication on the livers of rats was measured via the hepatic MDA level. Thiobarbituric acid (TBARS) assay was performed following Cayman protocol (Sigma, United States) and the spectrophotometer was read at 532 nm. Also, the antioxidant enzymes of liver cells, glutathione peroxidase (GPx), superoxide dismutase (SOD), and catalase (CAT) were evaluated taking the recommendation of Cayman Company (Sigma, United States). The results were read from the spectrophotometer at 450, 540, and 340 nm for SOD, CAT, and GPx, respectively.

### 2.9 Gross and histopathology studies

The rats’ livers were macroscopically examined for the presence of macronodules and micronodules followed by fixing liver samples in 10% formalin solution mixed with phosphate-buffered saline (pH 7.2). 24 hours post-fixation, the liver cassettes were processed in a paraffin tissue processing machine followed by embedding and sectioning of liver tissues at 5 µm thickness. Liver sections were kept in the incubator at 45°C for 48 h and then separated into two sets of sections. One set was stained with hematoxylin and eosin to study the changes in the liver architecture (Jayash et al., 2021), while the second set was stained in Masson’s Trichrome to study the grade of liver fibrosis via collagen fibers staining. The green-stained fibrotic areas were quantified using an image-processing program (Adobe Systems Inc., San Jose, CA, United States) after the steps of Jensen (Jensen, 2013).

### 2.10 Immunohistochemistry staining

For the immunohistochemistry study, liver sections were prepared in poly-L lysine slides and kept in the oven (Venticell, MMM, Einrichtungen, Germany) for 30 min at 45°C. The sections were then deparaffinzed using xylene and rehydrated gradually in different concentrations of alcohol. The concerned antigens of all tissue samples were retrieved in a microwave using pre-boiled sodium citrate buffer (10 mM) (EBsciences, United States) for 10 min. The method of staining was conducted as previously performed (Salama et al., 2013) following the protocol of Dakocytomation, United States. In short, a 3% peroxidase block reagent provided by the company was used as a blocking agent to the endogenous peroxidase for 5 minutes and then the liver sections were carefully treated with phosphate-buffered saline (PBS, pH 7.2) prepared fresh before use. Following buffer washing, the tissue sections were then incubated inside the humid chamber for 15 min with the different target antibodies. The concentrations of the antibodies used were optimized as TIMP-1 (1:50) (Abcam, United States), MMP-2 (1:50), and α-SMA (1:25) (Novusbio, England). Following washing, the tissue sections were covered with streptavidin-HRP and kept inside the humid chamber for 15 min. Diaminobenzidined (DAB) substrate chromagen was added to the liver tissues after washing and left incubated for an additional 5 min Gradual alcohol and xylene, the liver tissues were dehydrated, and finally covered and slipped for microscopic examination (Jayash et al., 2017). The stained tissues were quantitatively analyzed using an image-processing program (Adobe Systems Inc., San Jose, CA, United States) based on Jensen (Jensen, 2013).

### 2.11 Data analysis

Data analysis of the current study was operated statistically using the program of one-way ANOVA and the Tukey test in analyzing all data. All values are posted as the mean ± SD. The value of probability was set as significant at *p* ≤ 0.05.

## 3 Results

### 3.1 Azino-bis 3-ethylbenzthiazoline-6-sulfonic scavenging activity of Q-Br

The scavenging activity of the compound Q-Br to ABTS^+^ radical in comparison with the standards of ascorbic acid (AC) and gallic acid (GA) is illustrated in Figure 1. The scavenging capacity of Q-Br was significantly low compared to both standards, but remained at acceptable capacity.

### 3.2 *In vivo* hepatoprotective activity

#### 3.2.1 Liver index

The weight of the body and index of the liver tissues in the present sub-chronic liver damage study are displayed in Table 1. Although there was no significance in the initial body weight of all the rats, the final body weight recorded from the cirrhosis control group was considerably decreased in comparison with those of normal rats due to TAA toxicity. Additionally, the final rats’ body weight treated with LD Q-Br (25 mg/kg) and HD Q-Br (50 mg/kg) were considerably higher in comparison with the cirrhosis control group as a result of daily treatment of the rats with Q-Br that showed significant hepatoprotective potential. Contrarily, HD Q-Br did not record a significant difference when compared with those given the same dose of the standard drug Silymarin. The liver index measured from the cirrhosis control group was significantly higher when compared to normal (healthy) rats, while those treated with Silymarin and low- and high-dose Q-Br revealed a detectable decrease in the liver index in comparison with the cirrhosis control group. Furthermore, there was no significant difference between the liver indexes measured from the animals treated with HD Q-Br and Silymarin-treated ones.

**TABLE 1:** Effect of Q-Br treatment on the final body weight and liver index of the rats after 15 weeks of sub-chronic liver injury induced by TAA.

Group	Initial body weight	Final body weight	Liver weight	Liver index
	(g)	(g)	(g)	%
Cirrhosis control	268 ± 35.57	413 ± 29.03**	19 ± 1.04**	5 ± 0.18**
LD Q-Br	299 ± 6.13	455 ± 20.58*#	18 ± 0.99	4 ± 0.27*#
HD Q-Br	286 ± 36.61	493 ± 10.93*	18 ± 1.72	4 ± 0.22*
Reference control	275 ± 28.69	506 ± 19.87*	17 ± 0.52*	3 ± 0.07*
Normal control (healthy)	277 ± 22.41	499 ± 6.97	16 ± 2.22	3 ± 0.35

Cirrhosis control: TAA-treated rats; the LD Q-Br treated group (25 mg/kg); the HD Q-Br treated group (50 mg/kg); Silymarin-treated rats (50 mg/kg); normal (apparently healthy): liver from normal rat given 5% Tween 20; data are presented as Mean ± SD, while n = 6 and *p* ≤ 0.05 was significantly considered. *p < 0.001 vs. cirrhosis control group, #*p* < 0.05 vs. Silymarin-treated group and **p < 0.05 vs. normal group.

### 3.3 Liver biochemistry

The sub-chronic liver injury that was induced by TAA for 15 weeks in our study resulted in a significant elevation in the liver biochemistry parameters as shown in Table 2. Oral administration of LD Q-Br (25 mg/kg) and HD Q-Br (50 mg/kg) revealed remarkable (*p* < 0.05) attenuation in the serum level of liver cells’ antioxidant enzymes (AP, ALT, AST, GGT, and LDH) as well as serum bilirubin in comparison with the cirrhosis control group. With the exception of bilirubin, the data recorded from the high-dose Q-Br did not show significance in comparison with the reference control group that administered Silymarin (50 mg/kg).

**TABLE 2:** Effect of Q-Br treatment on the liver biochemistry of the rats after 15 weeks in sub-chronic liver injury induced by TAA.

Group	AP	ALT	AST	GGT	LDH	Bilirubin
	(IU/L)	(IU/L)	(IU/L)	(IU/L)	(IU/L)	(µmol/L)
Cirrhosis control	229 ± 8.57**	105 ± 7.22**	94 ± 5.99**	13 ± 1.42**	948 ± 68.00**	8 ± 0.46**
LD Q-Br	208 ± 12.25*#	91 ± 5.28*#	81 ± 10.69*#	6 ± 1.35*	754 ± 74.15*#	6 ± 0.57*#
HD Q-Br	200 ± 10.21*	88 ± 10.17*	79 ± 7.56*	5 ± 2.20*	516 ± 43.23*	5 ± 1.39*#
Reference control	191 ± 5.05*	78 ± 3.78*	68 ± 3.74*	4 ± 1.43*	457 ± 8.21*	3 ± 0.68*
Normal control	181 ± 8.62	74 ± 7.71	67 ± 3.45	3 ± 0.48	417 ± 33.01	3 ± 0.44

Cirrhosis control: TAA-treated rats; the LD Q-Br treated group (25 mg/kg); the HD Q-Br treated group (50 mg/kg); Rreference group: Silymarin-treated rats (50 mg/kg); normal (apparently healthy) control: liver from a normal rat; data are presented as Mean ± SD, while n = 6 and *p* ≤ 0.05 was significantly considered. *p < 0.05 vs. cirrhosis control group, #*p* < 0.05 vs. Silymarin-treated group and **p < 0.05 vs. normal group.

### 3.4 Liver macroscopy and imaging

**FIGURE 2:**
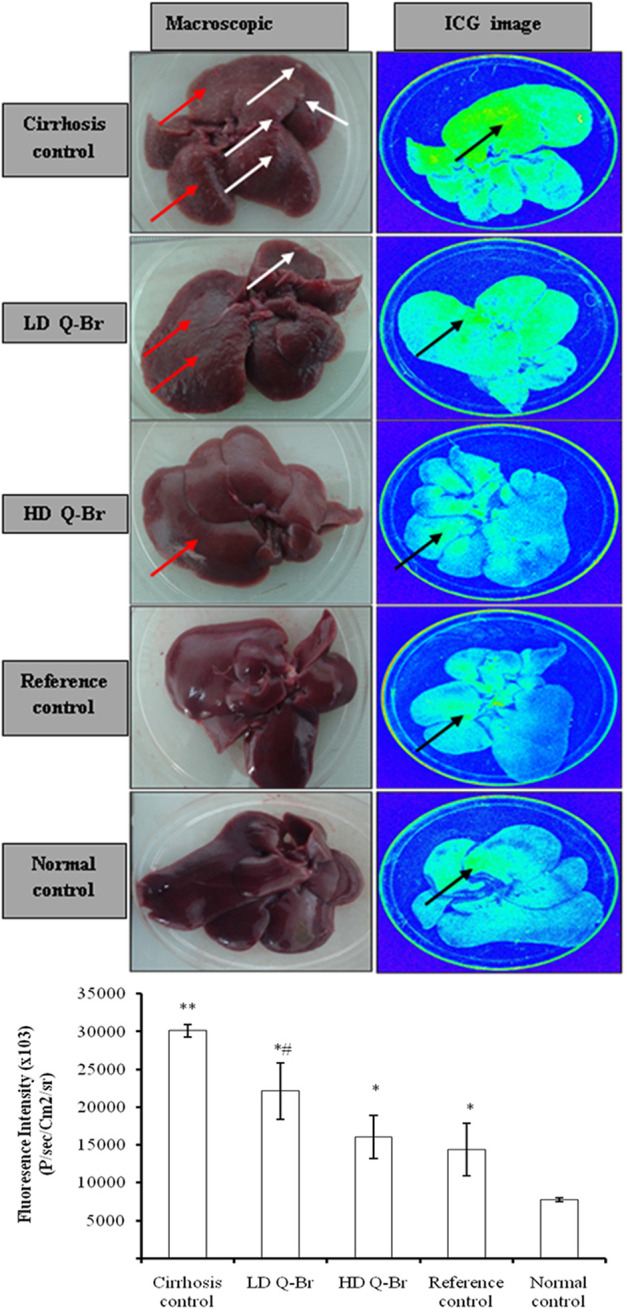
Impact of Q-Br treatment on the liver macroscopic and ICG fluorescent images in sub-chronic liver injury induced by TAA for 15 weeks study. Left column represents macroscopic pictures of livers from different experimental groups and the right column represents the ICG fluorescent images of the same livers of rats after intravenous injection of ICG (0.5 mg/kg). Cirrhosis control: TAA-treated rats; the LD Q-B-treated group (25 mg/kg); the HD Q-Br treated group (50 mg/kg); Silymarin-treated rats (50 mg/kg); Normal (apparently healthy): the liver from normal rat given 5% Tween 20. The white arrow represents macronodules of the cirrhotic liver, the red arrow represents micronodules, and the black arrow represents ICG-staining. The graph displays the ICG fluorescent intensity measured from the rats’ livers after intravenous injection of ICG bolus (0.5 mg/kg). The recorded measures are presented as Mean ± SD, while *n* = 3 and *p* ≤ 0.05 is significantly considered. **p < 0.05 vs. normal group; *p < 0.05 vs. cirrhosis control group; and ^#^ *p* < 0.05 vs. Silymarin-treated group.

The effect of the Q-Br treatment on the macroscopic liver architecture and the ICG fluorescent images of liver samples captured from the present sub-chronic study as well as the fluorescent intensity measured from all rats are displayed in Figure 2. Macroscopic results manifested that the untreated liver of the cirrhosis control group showed the formation of macro- and micro-nodules. Contrariwise, the liver samples collected from the LD Q-Br animals showed a significant reduction of macronodules, while those from HD Q-Br showed no signs of macro- or micro-nodules. The ICG image showed a high accumulation of ICG in the cirrhosis control liver, which is 3.8 fold higher in the fluorescent intensity compared to normal (7,813,000 ± 270,000 *P*/s/cm2/sr). The liver treated with the high dose Q-Br (50 mg/kg) displayed improvement in the macroscopic liver architecture more than the low dose liver (25 mg/kg). These results were confirmed by the less accumulation of ICG fluorescence which recorded 1.9 and 1.3 folds lower intensity in the high-dose and low-dose Q-Br-treated liver, respectively. Furthermore, the macroscopic picture and the ICG image from the high-dose liver showed comparable improvement to that of the Silymarin-treated liver which measured fluorescent intensity of 14,419,000 ± 3,469,000 *P*/s/cm^2^/sr) indicating proportional hepatoprotective activity of the high-dose Q-Br to that of the standard drug Silymarin.

### 3.5 Plasma indocyanine green clearance

**FIGURE 3:**
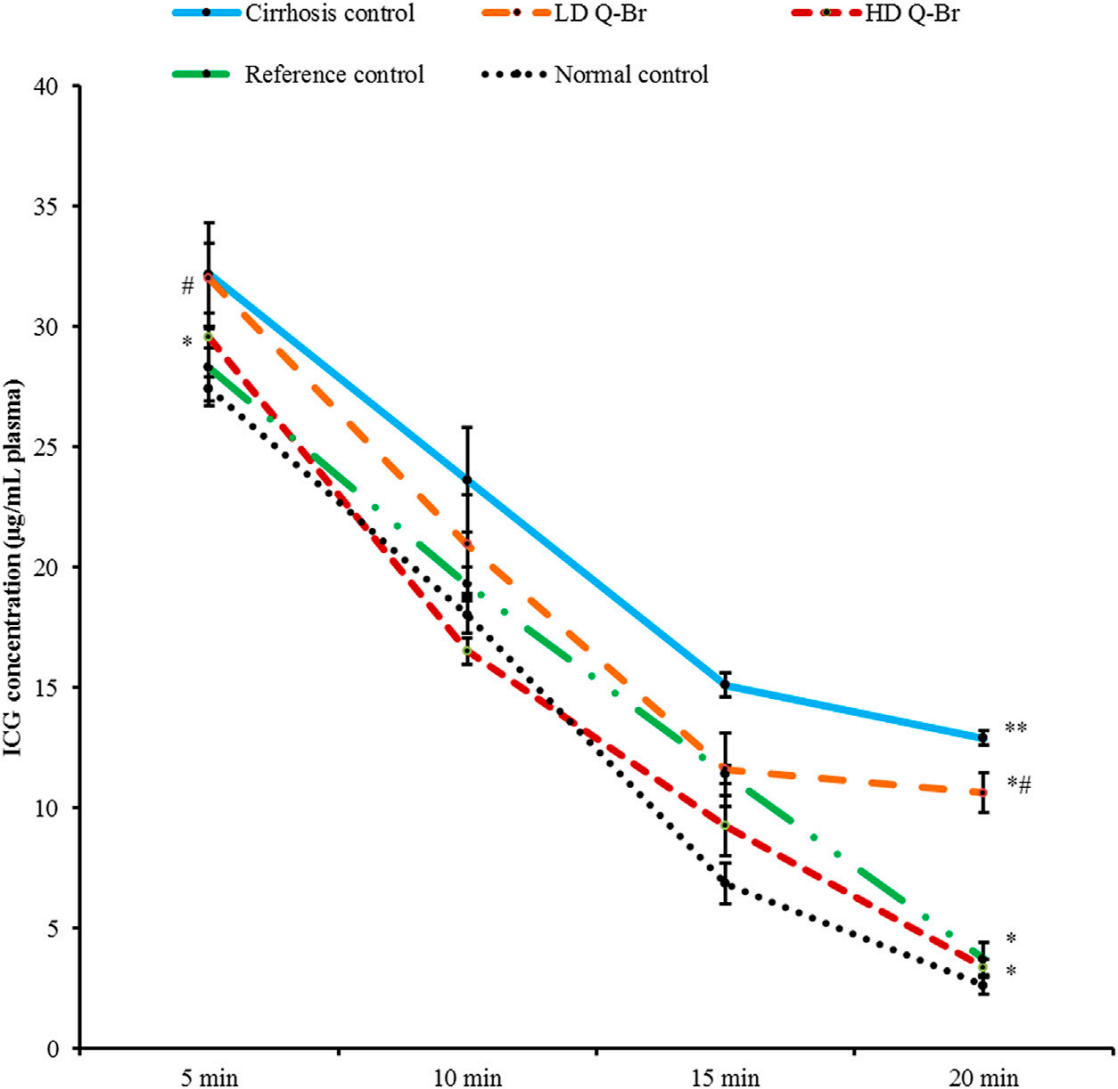
Plasma ICG concentration measured from all experimental groups at 5, 10, 15, and 20 min post intravenous injection of ICG bolus (0.5 mg/kg) into the tail vein. Cirrhosis control: TAA-treated rats; the LD Q-Br treated group (25 mg/kg); the HD Q-Br treated group (50 mg/kg). Silymarin-treated rats (50 mg/kg); Normal: the liver from normal rat given 5% Tween 20. Data are displayed as mean ± SD (*n* = 3) and *p* < 0.05 was considered significantly considered. **p < 0.05 vs. normal (healthy) group and *p < 0.05 vs. cirrhosis control group.

The plasma concentration of ICG measured from the rats at 5, 10, 15, and 20 min post-ICG injection into the tail vein of the rat is diagrammed in Figure 3. The data showed remarkably higher concentration in the cirrhosis control group reaching 12.90 ± 0.83 μg/ml plasma compared to the normal group (2.61 ± 0.29 μg/ml plasma) at 20 min and reflecting slow ICG clearance from the blood. Although the high dose Q-Br treatment (50 mg/kg) did not show significance 5 min after ICG injection (29.56 ± 0.50 μg/ml plasma) compared to the cirrhosis control group (32.19 ± 1.44 μg/ml plasma), but the plasma concentration of ICG was gradually and markedly reduced at 10 and 15 min reporting 3.37 ± 0.35 μg/ml plasma 20 min after ICG injection. In addition, the ICG plasma clearance recorded from the high dose Q-Br at 5, 10, 15, and 20 min did not reveal any significance compared to the Silymarin-treated group. Moreover, the data recorded from the high dose Q-Br at 20 min approached that from the reference control group treated with Silymarin (3.71 ± 0.33 μg/ml plasma) at the same time. Treatment of the rats with the low-dose Q-Br (25 mg/kg) did not record significance in ICG plasma concentration compared to cirrhosis control or silymarin-treated groups.

### 3.6 Hepatic antioxidant enzymes and malondialdehyde

The boosting effect of Q-Br on the liver endogenous enzymes is tabulated in Table 3. Thioacetamide administration to the rats remarkably elevated the MDA level in the liver homogenate of cirrhosis control rats. The Q-Br treatment has notably altered the damaging effect of TAA. On the reverse, the collapsed activity of hepatic antioxidant enzymatic candidates (SOD, CAT, and GPx) caused by TAA was distinctly elevated with the Q-Br treatment and without significant difference between HD Q-Br and Silymarin-treated groups.

**TABLE 3:** Impact of Q-Br treatment on the liver MDA level and antioxidant enzymatic activity of the rats after 15 weeks in sub-chronic liver injury induced by TAA.

Group	MDA	SOD	CAT	GPx
	nmol/mg protein	U/mg protein	nmol/min/mg protein	nmol/min/mg protein
Cirrhosis control	8.13 ± 0.12**	13.13 ± 0.97**	31.82 ± 1.16**	266.67 ± 6.43**
LD Q-Br	4.33 ± 0.13*#	24.37 ± 0.48*#	57.00 ± 3.43*#	319.59 ± 7.51*#
HD Q-Br	2.17 ± 0.03*	30.50 ± 0.34*	90.42 ± 3.73*	457.71 ± 20.97*
Reference control	2.02 ± 0.12*	30.55 ± 1.04*	93.76 ± 0.33*	468.77 ± 4.93*
Normal control	0.97 ± 0.05	32.22 ± 0.73	91.67 ± 1.94	483.37 ± 13.81

Cirrhosis control: the TAA-treated rats; the LD Q-Br treated group (25 mg/kg); the HD Q-Br treated group (50 mg/kg); reference group: the Silymarin-treated rats (50 mg/kg); normal control (healthy): liver from a normal rat; data are displayed as Mean ± SD, while n = 6 and *p* ≤ 0.05 was significantly considered. *p < 0.05 vs. cirrhosis control group, #*p* < 0.05 vs. The Silymarin-treated group and **p < 0.05 vs. normal group.

### 3.7 Histopathology analysis

**FIGURE 4:**
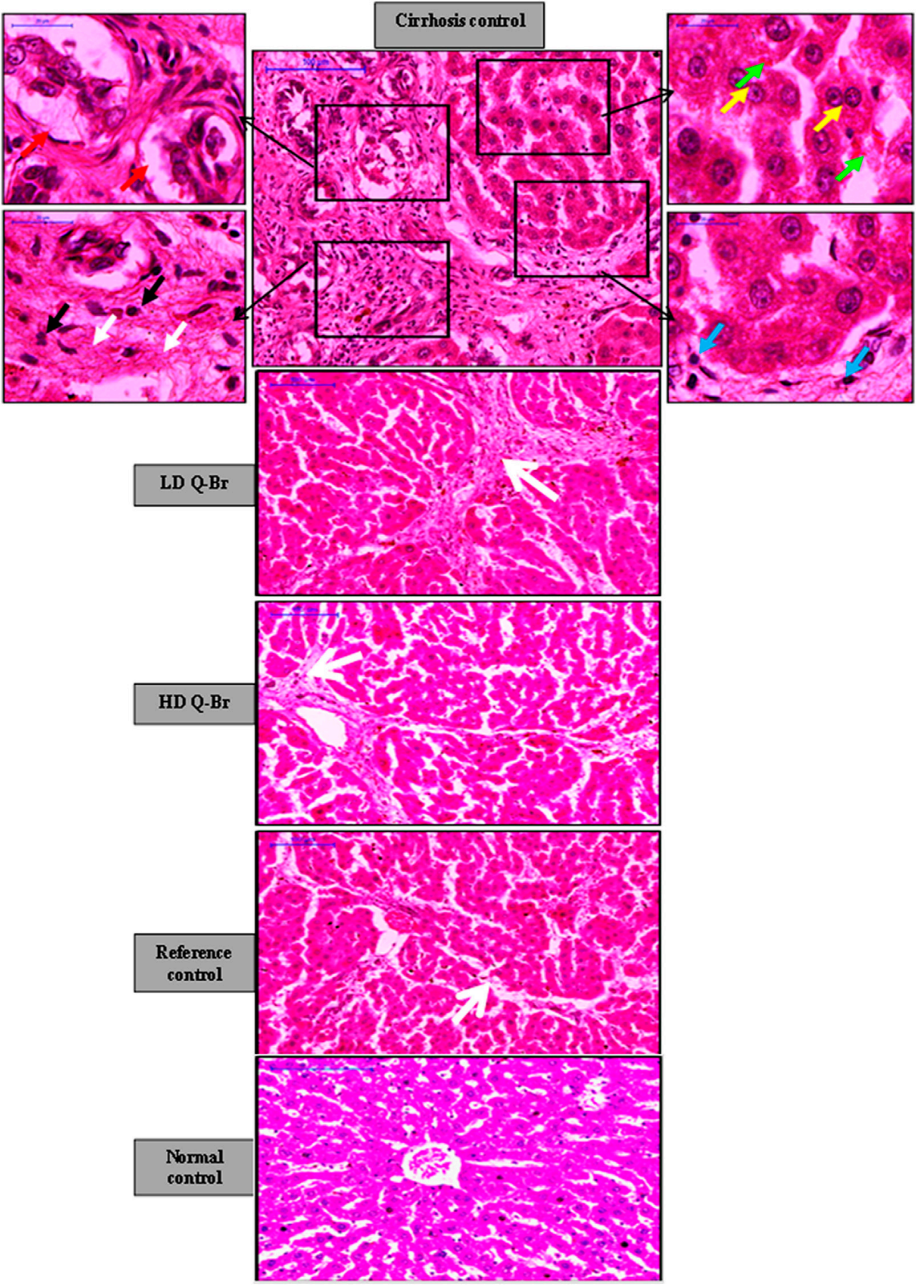
Hepatoprotective effect of Q-Br on the liver histpathology of hematoxylin and Eosin (H and E) staining showing gradual improvement in the fibrotic status of the rats’ livers. The cirrhosis control group liver treated with thioacetamide (TAA) revealing fibrotic septae extending from the central vein (white arrow), intensive mononuclear inflammation (blue arrow) and neutrophil infiltration (black arrow), bile duct proliferation (red arrow), binucleated (yellow arrow), and apoptotic hepatocytes (green arrow). Significant gradual reduction in the formation of fibrotic septae, neutrophil infiltration along with absence of bile duct proliferation is noticeable from the LD Q-Br (25 mg/kg) to the HD Q-Br (50 mg/kg) to the Silymarin-treated group (50 mg/kg), respectively.

The liver sections’ histology from all rats is illustrated in Figure 4. Hematoxylin and eosin staining showed progressive improvement in the liver histology of LD Q-Br (25 mg/kg) and HD Q-Br (50 mg/kg) in comparison with the TAA control group animals. Injecting the animals with a thioacetamide dose of 200 mg/kg three times weekly has led to the formation of fibrotic septae spreading from the central vein and replacing the apoptotic hepatocytes. Additionally, binucleated hepatocytes, mononuclear inflammation, neutrophil infiltration, and bile duct proliferation were obviously detectable in the cirrhosis control group. Oral administration of the Q-Br compound has dose-dependently improved the histology of the liver, while the HD Q-Br (50 mg/kg) showed better hepatoprotective activity than LD Q-Br (25 mg/kg) and approached that of Silymarin-treated rats at a similar dose (50 mg/kg).

**FIGURE 5:**
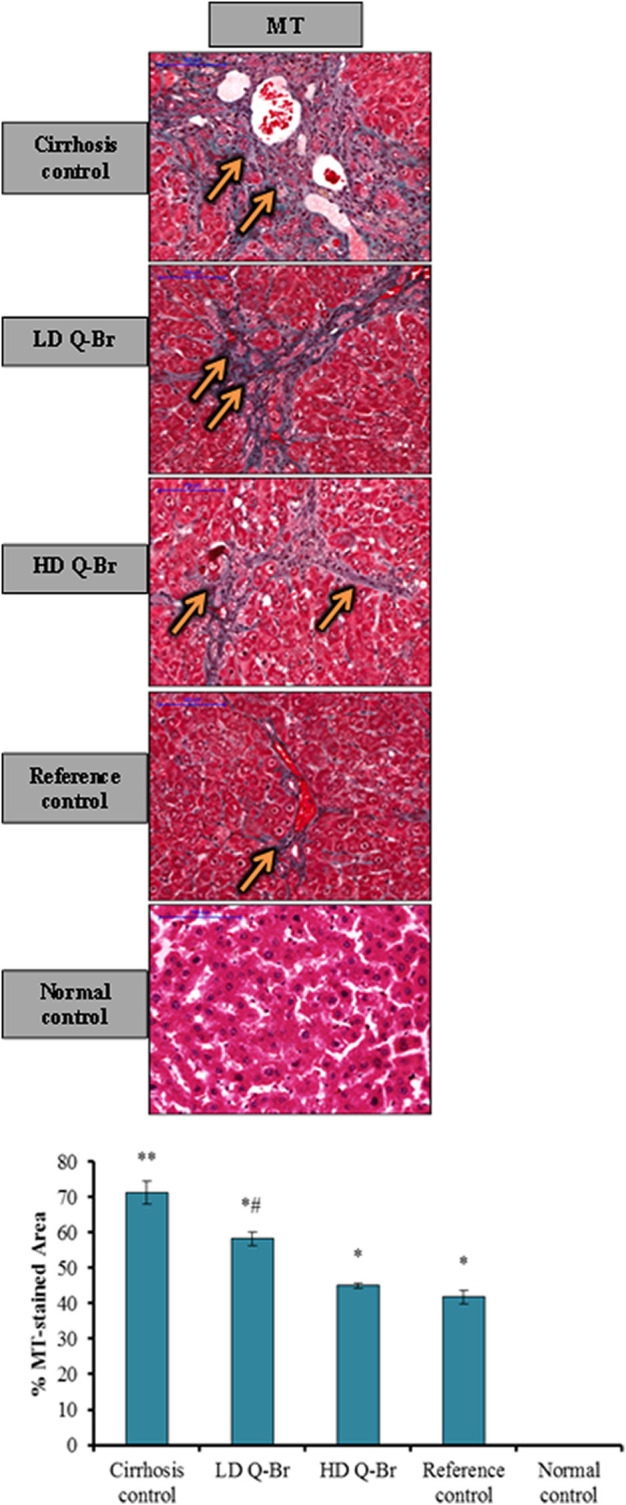
Q-Br treatment impact on Masson’s Trichrome staining (MT) is showing intensive green staining of collagen fibers (orange arrow) in the cirrhosis control group. Considerable reduction of MT staining from LD Q-Br to HD Q-Br and Silymarin-treated livers, respectively. Scale bar indicates 100 µm magnifications. The quantitative analysis of MT staining of liver tissue sections is diagrammed below the images. All the data are expressed as Mean ± SD, *n* = 3 and *p* is significantly considered at value ≤ 0.05. **p < 0.05 vs. normal group; *p < 0.05 vs. cirrhosis control group; and ^#^ *p* < 0.05 vs. Silymarin-treated group. Immunohistochemistry of fibrotic and extracellular matrix proteins.

Masson’s Trichrome stain displayed a significant gradual reduction of the specific green stain of collagen fibers in the liver sections of LD Q-Br, HD Q-Br, and Silymarin-treated animals in comparison with cirrhosis control animals. Quantification of the green-stained fibrotic areas in the Masson’s Trichrome images confirmed the qualitatively-stained images of the Q-Br treatment compared to the other groups (Figure 5).

**FIGURE 6:**
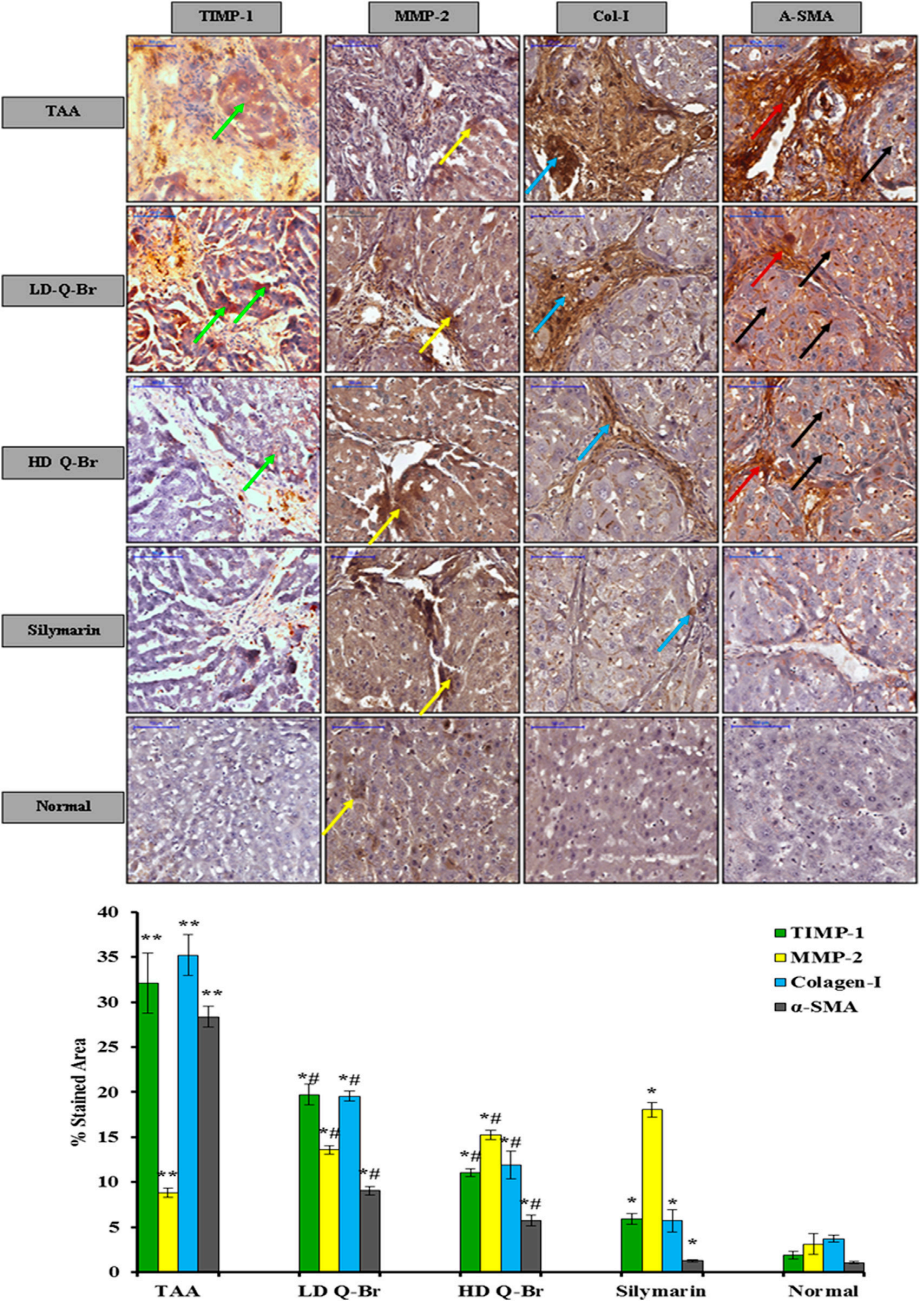
Q-Br treatment impact on the extracellular matrix protein family, tissue inhibitor of metalloproteinase (TIMP-1), metalloproteinase (MMP-2), and the hepatic stellate cells marker, alpha-smooth muscle actin (α-SMA). Noticeable downregulation of TIMP-1 (green arrow), myofibroblasts (red arrow), and α-SMA (black arrow) staining in the livers of rats treated with the low-dose (LD Q-Br, 25 mg/kg), high-dose (HD Q-Br, 50 mg/kg), and Silymarin (50 mg/kg) in comparison with that of the TAA control group which shows upregulation of the target antigens. On the other hand, significant upregulation of MMP-2 (yellow arrow) is obviously expressing in the liver tissues of rats treated with the low-dose (LD Q-Br, 25 mg/kg), high-dose (HD Q-Br, 50 mg/kg), and Silymarin-treated livers (50 mg/kg) in comparison with that of the TAA control group which showed significant downregulation of MMP-2. Scale bar indicates 100 µm magnifications. Quantitative analysis of immuno-stained markers (TIMP-1, MMP-2 and α-SMA) of the liver sections obtained from all animal groups is outlined below the related photographs. All the data are expressed as Mean ± SD, *n* = 3 and *p* is significantly considered at value ≤ 0.05. **p < 0.05 vs. normal group; *p < 0.05 vs. cirrhosis control group; and ^#^ *p* < 0.05 vs. Silymarin-treated group.

The impact of Q-Br administration on the hepatic stellate cells indicator α-SMA and extracellular protein indicators (TIMP-1 and MMP-2) in the liver tissue sections stained from all animal groups is displayed in Figure 6. Microscopic examination claimed remarkable downregulation of TIMP-1, Col-I, and α-SMA in the rats’ livers treated with Q-Br in a dose-responsive manner compared to the noticeable downregulation of the same immune-stained proteins from the cirrhosis control. On the other hand, Q-BR exposed significant upregulation in the stained metalloproteinase protein MMP-2 compared to the cirrhosis control group which recorded reversed results. Furthermore, the immune-staining obtained from the HD Q-Br liver was correspondent to that obtained from the Silymarin-treated group. Qualitative immunohistostaining of liver sections was confirmed quantitatively as shown in Figure 6.

## 4 Discussion

Medications with side effects on the liver have shown extensive use in modernized life today and as a result, liver cirrhosis has become a significant problem (Asrani et al., 2019). Therefore, this experiment targeted on finding new therapeutic remedies to reduce liver cirrhosis. Quinazoline-based compounds showed many biological activities as mentioned earlier encouraging more studies on these compounds to find alternative therapy for liver diseases with more efficacies and fewer side effects.

Accumulation of reactive oxygen species (ROS) resulting from injured hepatocytes play important role in fibrogenesis and carcinogenesis (Sakurai et al., 2013). Therefore, exogenous antioxidant compounds can contribute to alleviating the ROS load of the fibrotic liver along their scavenging capacity. The current study evaluated the antioxidant capacity of Q-Br, a quinazoline Shiff base compound. Although some quinazoline derivatives measured antioxidant capacity higher than ascorbic acid (Soliman et al., 2020), Q-Br compound revealed ABTS scavenging capacity three-folds less than ascorbic acid indicating low free radical scavenging capacity which is perhaps sufficient enough for performing its antioxidant task. The results of the present study are consistent with the publication of Shang et al., 2018 who mentioned that the quinazoline derivative Tryptanthrin showed significantly lower antioxidant capacity compared to the standard trolox, but it recorded hepatoprotective activity to experimentally-induced hepatic cell toxicity via the blockage of reactive oxygen species production upon the administration of the insult (Shang et al., 2018).

Next, we evaluated the *in vivo* hepatoprotective potential of Q-Br in sub-chronic liver injury by TAA in rats. The significant improvement in the liver weight and the considerable restoration of liver function test of the animals treated with Q-Br is consistent with another study that showed the attenuating effect of a quinazoline alkaloid on the CCl4-induced destructed liver enzymes (Sarkar et al., 2014). The Q-Br treatment regimen has positively and dose-dependently induced the improvement in the architectural, histological, and functional status of the damaged liver. These results were confirmed by the limited binding of ICG to the protein molecules of the liver and the decreased fluorescent intensity measured from the Q-Br-treated rats. Furthermore, the hepatoprotective potential of Q-Br was evidenced by the accelerated clearance of ICG from the blood plasma of the Q-Br-treated rats. Similarly, Mathes *et al.* (Mathes et al., 2008) used an ICG plasma clearance test to measure liver function in his rat model of hepatoprotection. In a recent study, Chen et al., 2021 stated that the ICG inflorescence test could successfully estimate the liver damage status in CCl4-induced liver damage in experimental rats (Chen et al., 2021).

Oxidative stress was proved to be one of the main reasons for liver damage induced by TAA in experimental animals (Abood et al., 2020; Elnfarawy et al., 2020). Progression of liver damage by TAA releases free radicals which play a pivotal role in reaching cirrhosis status (Mustafa et al., 2013). For several decades, MDA has been used as an indicator for lipid peroxidation in damaged tissues including liver injury (Nielsen et al., 1997; Cengiz et al., 2020). Studies reported that antioxidant enzymes play crucial role in protecting the liver from the damage induced by chemicals (Choi et al., 2021). In our study, oral administration of Q-Br to the rats along with TAA intoxication for 15 weeks study has restored the oxidant/antioxidant balance of the liver through the inhibition of lipid peroxidation and enhancement of the production of endogenous antioxidant enzymes of hepatocytes. Reportedly, scientists showed the hepatoprotective activity of quinazoline derivatives via their enhancement activity of hepatocellular antioxidant enzymes (Kusuma and Vedula, 2016).

Adaptive and innate host immune responses play a crucial role in liver injury induced by toxins and drugs (Yokoi and Oda, 2021). The current study revealed inflammatory infiltrates of neutrophils into the fibrotic area of the TAA-injured liver as previously reported (Salama et al., 2012). Furthermore, the reduced collagen content measured qualitatively and quantitatively by Masson’s Trichrome staining from Q-Br-treated liver sections confirms the restoration of the normal liver architecture. Similarly, Sarkar *et al.* (Sarkar et al., 2014) noticed the improvement of liver histology by quinazoline alkaloids against chemically induced liver injury in rats. In parallel to the present study, Borik and Hussein have recently conducted a docking study on Novel quinazoline derivatives and concluded that these compounds suggested hepatoprotective activity against lipopolysaccharide-induced liver damage through their alteration of biomarkers of oxidative stress (Borik and Hussein, 2022b).

The main feature of chronic damage to the liver is the deposition of ECM proteins in liver tissues. The fibrogenesis process is resolved by the discharge of TGF-β1 from the endothelial cells, activated HSCs, and kupffer cells. TGF-β1 is considered a critical pro-fibrogenic mediator that enhances the production of tissue inhibitors of metalloproteinase (TIMPs), inhibits the degradative matrix metalloproteinase enzymes (MMPs), and increases the synthesis of collagens, proteoglycans, and fibronectin (Scott et al., 2015). Repeated damage to the liver is thought to induce the differentiation of activated HSCs into myofibroblasts which excessively synthesize α-SMA, collagen type I and III (Poonkhum et al., 2011). Therefore, inhibition of the HSCs strategy is considered a pharmaceutical target in liver disorders (Xu et al., 2015). The suppressed activity of Q-Br to the α-SMA expression and restoration of the balance between the activity of TIMP-1 and MMP-2 in favor of tissue repair can be attributed to the reduced activity of HSCs and accordingly the level of the pro-fibrogenic mediator TGF-β1. Likewise, Liang *et al.* (Liang et al., 2013) attributed the hepatoprotective efficacy of the quinazolie alkaloid halofuginone to its anti-fibrotic activity. The limitations of this study are few references used in the discussion to confirm the results of the present study due to the few studies conducted on the hepatoprotectivity of quinazoline compounds and further studies need to be conducted on the same compound using other animal models and other insults to induce liver damage.

## 5 Conclusion

This study presents novel research findings that scientifically demonstrate that the synthesized compound Q-Br plays a protective role against liver injury. Concomitantly, the quinazoline derivative Q-Br exhibited hepatoprotective therapy against sub-chronic TAA hepatotoxicity via restoration of the normal liver status as confirmed macroscopically and ICG bio-imaging study showing fast clearance of ICG from the liver. The biochemistry analysis revealed recovery of the specific liver markers as well as oxidative stress parameters. The histopathology study displayed noticeable improvement of the liver architecture. Immunohistochemistry screening suggested remarkable anti-fibrotic activities. Taken together, these results suggest promising therapy for inhibiting the progress of liver fibrosis. Therefore, Q-Br merits further studies on its hepatoprotective activity down to the cellular level.

## Data Availability

The original contributions presented in the study are included in the article/Supplementary Material; further inquiries can be directed to the corresponding authors.
